# The association between restless legs syndrome and chronic venous insufficiency: a systematic review

**DOI:** 10.25122/jml-2025-0075

**Published:** 2025-10

**Authors:** Malak Abdullah Alyahya, Raghad Almansour, Alhanouf Altamimi, Norah AlAkeel

**Affiliations:** 1 Department of Family Medicine, King Abdulaziz Medical City, Ministry of the National Guard Health Affairs, Riyadh, Kingdom of Saudi Arabia; 2 Department of Family Medicine, King Fahad Medical City, Riyadh Second Health Cluster, Riyadh, KSA

**Keywords:** restless leg syndrome, chronic venous insufficiency, superficial venous reflux, varicose veins, risk factors

## Abstract

Restless legs syndrome (RLS) and its association with venous disorders have garnered attention in medical literature. This systematic review aims to consolidate current evidence on the relationship between RLS and various venous pathologies, exploring potential mechanisms, interventions, and clinical implications. A comprehensive search of electronic databases identified relevant studies published up to January 2024. Inclusion criteria comprised studies investigating the association between RLS and venous disorders, encompassing a diverse range of methodologies. Data extraction and quality assessment were performed to ensure the robustness of the included studies. The systematic review included studies that explored associations between RLS and conditions such as superficial venous reflux, varicose veins, and chronic venous insufficiency. Findings from Dezube *et al*. and Pyne *et al*. indicated a positive correlation between RLS and superficial venous pathologies, with interventions such as superficial venous ablation and ultrasound-guided foam sclerotherapy showing promising outcomes. Sundaresan *et al*. extended the exploration to leg vein treatments, reporting improvements in RLS symptoms post-intervention. These results underscore the complexity of the relationship between RLS and venous disorders. The systematic review provides an overview of the current evidence on the association between RLS and various venous pathologies. The positive correlations observed in some studies suggest a potential role for addressing underlying venous pathology in managing RLS symptoms. However, the heterogeneity in study designs and outcomes calls for further research to elucidate the underlying mechanisms and refine targeted interventions.

## Introduction

Restless legs syndrome (RLS) and chronic venous insufficiency (CVI) represent two distinct yet potentially interconnected neurological and vascular conditions that have garnered significant attention in medical literature [1-3]. RLS is characterized by an irresistible urge to move the legs, often accompanied by uncomfortable sensations, particularly during periods of rest or inactivity [4,5]. On the other hand, CVI results from impaired venous blood flow, leading to venous hypertension and subsequent symptoms such as leg swelling, pain, and skin changes [6-8].

While RLS and CVI have traditionally been studied in isolation, emerging evidence suggests a potential association between these two conditions [9,10]. The rationale for investigating this relationship lies in the shared anatomical and physiological aspects of the lower extremities, where both RLS symptoms and CVI manifest. The intricate network of veins, arteries, and neural pathways in the legs provides a complex substrate for the potential intersection of these two disorders.

This systematic review aims to comprehensively examine the existing body of literature to elucidate the nature of the association between RLS and CVI. Understanding this potential link is crucial for several reasons. Firstly, it may provide valuable insights into the underlying pathophysiological mechanisms of both conditions, shedding light on shared etiological factors or common triggers. Secondly, recognizing a possible association may have implications for the clinical management of patients presenting with symptoms of either RLS or CVI, prompting a holistic approach to assessment and treatment.

The scarcity of systematic investigations exploring the interplay between RLS and CVI underscores the need for a comprehensive review. By synthesizing available evidence, this review aims to identify patterns, trends, and gaps in the current literature, ultimately guiding future research directions. Moreover, a nuanced understanding of the relationship between RLS and CVI may contribute to the development of targeted therapeutic interventions that address both neurological and vascular aspects, thereby improving the overall quality of life for affected individuals.

## Material and methods

This systematic review was conducted following established guidelines outlined in the Preferred Reporting Items for Systematic Reviews and Meta-Analyses (PRISMA) statement to ensure transparency, rigor, and reproducibility. The systematic search was conducted across multiple electronic databases, including PubMed, Embase, Scopus, and the Cochrane Library, from inception to the most recent update available at the time of the search (knowledge cutoff date: January 2024).

The search strategy was meticulously crafted using a combination of relevant Medical Subject Headings (MeSH) terms, keywords, and Boolean operators. The key search terms included variations of ‘restless legs syndrome,’ ‘chronic venous insufficiency,’ and associated synonyms. To enhance the comprehensiveness of the search, manual searches of reference lists of relevant articles, reviews, and conference proceedings were also conducted.

Inclusion and exclusion criteria were established as a priori to guide the selection process. Studies eligible for inclusion were required to meet the following criteria: (1) original research articles, (2) written in English, (3) investigating the association between restless legs syndrome and chronic venous insufficiency, (4) including human subjects, and (5) providing clear and relevant outcome measures. Studies involving animal models, case reports, editorials, and non-original research articles were excluded from the analysis.

Three independent reviewers conducted the initial screening of titles and abstracts to identify potentially relevant studies. Subsequently, the full texts of the selected articles were thoroughly reviewed to assess their eligibility based on the predefined criteria. Any disagreements were resolved through consensus or consultation with a fourth reviewer.

Data extraction was performed using a standardized form encompassing key study characteristics, including study design, sample size, participant demographics, diagnostic criteria for RLS and CVI, and relevant outcomes. The risk of bias within individual studies was evaluated using appropriate tools such as the Newcastle-Ottawa Scale for observational studies or the Cochrane Risk of Bias tool for randomized controlled trials.

## Results

**Figure 1:**
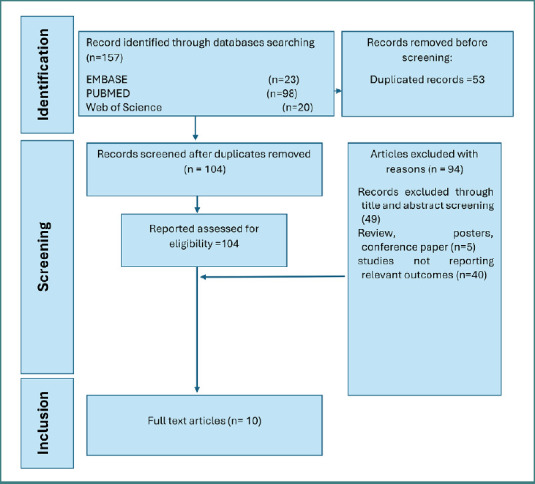
PRISMA flowchart depicting the study selection process

A total of 157 records were identified across electronic databases. After removing 53 duplicates, 104 studies were screened for eligibility. Of these, 94 were excluded for reasons detailed in Figure 1, resulting in 10 studies being included in the final analysis. Figure 1 depicts the PRISMA flow diagram outlining the study selection process.

Table 1 presents an overview of the general characteristics of the studies included in this systematic review. Among these, Dezube *et al*. investigated the correlation between RLS and SVR in 207 patients, with a notable association present [11]. Pyne *et al*. 2023 explored the association of varicose veins with RLS and nocturnal leg cramps in a sample of 506 individuals, with 209 of them showing the association [10]. Sundaresan *et al*. conducted a retrospective review on the treatment of leg veins for RLS in 134 patients, and the study reported an association [12]. In contrast, Yetkin *et al*. examined venous leg symptoms in patients with peripheral varicose veins (PVV) in a multicenter study involving 1,319 participants but found no significant association [13]. In general, the sample size among the studies ranged between 70 participants to 1,319 participants. Overall, 7 of the 10 studies reported a positive relationship between RLS and chronic venous insufficiency. Table 2 focuses on demographic factors across the included studies. Dezube *et al*. included patients below 18 years of age with signs or symptoms of venous reflux disease [11], whereas Pyne *et al*. incorporated individuals presenting with subjective symptoms of chronic superficial venous insufficiency (CSVI) [10]. Sundaresan *et al*. observed patients ranging from 38 to 81 years, predominantly females above 60 years [12], while Yetkin *et al*. admitted patients to outpatient clinics in Turkey for various reasons [13]. Yolgösteren *et al*. conducted a study on 541 patients diagnosed with RLS via polysomnography [14]. McDonagh *et al*. included subjects reporting to a phlebology group practice [15]. Acir *et al*. examined patients presenting with leg pain and cramps to the cardiovascular surgery outpatient clinic [16]. Fronek *et al*. focused on subjects reporting nocturnal leg symptoms, predominantly females [17]. Ulaş *et al*. studied 70 participants diagnosed with idiopathic RLS, excluding those below 18 or over 70 years, among other criteria [18]. Hayes *et al*. screened 89 patients with restless legs and subsequently selected 35 based on specific criteria [1]. Table 3 provides insights into the interventions and outcomes of the included studies. Dezube *et al*. implemented superficial venous ablation, resulting in 85.9% RLS symptom resolution and 94.4% successful resolution of reflux [11]. Pyne *et al*. used ultrasound-guided foam sclerotherapy (USGFS), demonstrating high technical and clinical success rates with symptomatic relief up to one year [10]. Sundaresan *et al*. applied radiofrequency ablation (RFA) of refluxed saphenous veins and ultrasound-guided foam sclerotherapy (UGFS), reporting improved RLS symptoms after correcting SVR [12]. Yolgösteren *et al*. focused on investigating chronic venous insufficiency in patients with sleep disorders due to RLS [14]. McDonagh *et al*. found a higher prevalence of cramps in RLS-positive subjects with concurrent CVD [15]. Acir *et al*. performed surgery, reporting that 63% of patients experienced improved sleep quality after varicose vein surgery [16]. Ulaş *et al*. observed a higher serum irisin level in idiopathic RLS cases compared to controls [18]. Hayes *et al*. compared operative and non-operative cohorts, finding a statistically significant improvement in symptoms after operative correction of superficial venous insufficiency [1].

**Table 1:** General characteristics of included studies

Author(s)	Year	Title	Total subjects (Association)	Association present
**Dezube A. *et al***. [11]	2021	Correlation between Restless Leg Syndrome and Superficial Venous Reflux	207	Yes
**Pyne R *et al***. [10]	2023	Lateral Sub dermic Venous Plexus Insufficiency: The Association of Varicose Veins with Restless Legs Syndrome and Nocturnal Leg Cramps	506 (209)	Yes
**Sundaresan S *et al***. [12]	2019	Treatment of Leg Veins for Restless Leg Syndrome: A Retrospective Review	134 (34)	Yes
**Yetkin E *et al***. [13]	2021	Venous leg symptoms, ecchymosis, and coldness in patients with peripheral varicose vein: A multicenter assessment and validation study (VEIN-VIOLET study)	1319	No
**Yolgösteren A *et al***. [14]	2020	Investigation of chronic venous insufficiency in patients with sleep disorders due to restless legs syndrome	541 (20/40)	-
**McDonagh B *et al***. [15]	2007	Restless legs syndrome in patients with chronic venous disorders: an untold story	174 (63)	Yes
**Acir I *et al***. [16]	2023	The implications of varicose vein surgery on sleep evaluation scales	160 (63%)	Yes
**Fronek L *et al***. [17]	2017	Nocturnal leg symptoms are not associated with specific patterns of superficial venous insufficiency	371 (0)	No
**Ulaş S *et al***. [18]	2023	Investigation of the relationship between serum irisin level in the idiopathic restless legs syndrome: Could be a marker independent of physical activity?	70 (0)	Yes
**Hayes C *et al***. [1]	2008	The effect of endovenous laser ablation on restless legs syndrome	89 (35)	Yes

**Table 2:** Demographic factors of included studies

Author(s)	Year	Age range	Gender distribution	Inclusion criteria	Exclusion criteria
**Dezube A. *et al***. [11]	2021	-	-	All patients less than 18 years of age presenting with signs or symptoms of venous reflux disease	Patients with no evidence of venous diseases
**Pyne R *et al***. [10]	2023	52.5	84% women	Patients presenting with subjective symptoms of CSVI and undergoing comprehensive venous ultrasound (CVU)	Cosmetic spider vein treatments without symptoms, isolated deep venous problems without focal leg symptoms
**Sundaresan S *et al***. [12]	2019	38-81	Typical RLS patient: Female above 60 years	-	-
**Yetkin E *et al***. [13]	2021	-	-	Admitted to outpatient cardiology and cardiovascular surgery clinics in Turkey	Regular analgesic users, K vitamin antagonists, oral anticoagulant users, history of various medical conditions
**Yolgösteren A *et al***. [14]	2020	-	-	Polysomnography performed due to sleep disorder	-
**McDonagh B *et al***. [15]	2007	-	Women	Subjects reporting to a phlebology group practice	-
**Acir I *et al***. [16]	2023	48.7	68.1% female	Patients presenting to Cardiovascular Surgery outpatient clinic with leg pain and cramps and diagnosed with venous insufficiency	Below 18 years old, history of deep vein thrombosis, symptomatic peripheral artery disease, pre-existing restless legs syndrome diagnosis, non-compliance with post-operative follow-up
**Fronek L *et al***. [17]	2017	~56	85% female	-	-
**Ulaş S *et al***. [18]	2023	27-61	68.6% female	Diagnosed with idiopathic RLS, aged between 18 and 70	Under 18 or over 70 years old, history of anemia, diabetes, peripheral neuropathy, peripheral artery disease, chronic renal failure, pregnancy, physical/mental disabilities
Hayes C *et al*. [1]	2008	49.4-58.8	Operative: 31.6% male, 68.4% female; non-operative: 6.3% male, 93.7% female	Restlessness in legs, meeting 2003 NIH criteria for RLS, IRLS score of 15 or greater	Conditions mimicking RLS, unrelated medical reasons for withdrawal

**Table 3:** Intervention and outcomes of included studies

Author(s)	Year	Intervention	Outcome
**Dezube A. *et al***. [11]	2021	Superficial venous ablation	85.9% RLS symptom resolution, 94.4% successful resolution of reflux, various outcomes post ablation
**Pyne R *et al***. [10]	2023	Ultrasound-guided foam sclerotherapy (USGFS)	High technical and clinical success rates, symptomatic relief up to 1 year
**Sundaresan S *et al***. [12]	2019	Radiofrequency ablation (RFA) of refluxed saphenous veins, USGFS of associated tributaries	Improved RLS symptoms after correcting SVR
**Yetkin E *et al***. [13]	2021	Not applicable	No significant association with PVV group
**Yolgösteren A *et al***. [14]	2020	Not applicable	Not applicable
**McDonagh B *et al***. [15]	2007	Not applicable	Higher prevalence of cramps in RLS-positive subjects with concurrent CVD, advanced form of CVD
**Acir I *et al***. [16]	2023	Surgery	63% reported improved sleep quality after varicose vein surgery
**Fronek L *et al***. [17]	2017	Not applicable	No significant difference between patterns of superficial venous reflux in patients with and without nocturnal symptoms
**Ulaş S *et al***. [18]	2023	Not applicable	Higher serum irisin level in idiopathic RLS cases compared to controls
**Hayes C *et al***. [1]	2008	Surgery (Operative) vs non-operative	Operative correction of SVI decreased mean IRLS score by 21.4 points, 80% improvement in symptoms

## Discussion

The comprehensive analysis of the selected studies elucidates the intricate interplay between restless legs syndrome and various venous disorders, offering valuable insights into potential mechanisms, clinical implications, and avenues for future research. The association between RLS and venous disorders has been a subject of increasing interest, and the findings of this systematic review align with previous research highlighting the bidirectional relationship between these conditions. Studies by Dezube *et al*. and Pyne *et al*. provide compelling evidence supporting a positive correlation, emphasizing the importance of addressing underlying venous pathology to alleviate RLS symptoms [10,11]. This aligns with the broader literature, where venous insufficiency has been implicated in the pathogenesis and exacerbation of RLS symptoms [19–21].

To elaborate further, Pyne *et al*. noted a correlation between CVI and RLS, specifically a strong association between RLS and the lateral subdermic venous plexus (LSVP), a venous network on the lateral thigh and calf which is often overlooked when evaluating varicose veins. In this study, RLS had a prevalence of 84% in 506 patients presenting with CVI symptoms. Venous ultrasound confirmed reflux in 85-92% of LSVP in patients presenting with RLS; conversely, patients reporting neither symptom had a high rate of negative LSVP reflux. Ultrasound-guided foam sclerotherapy (USGFS) treatment of these LSVP varicose veins led to 93% of patients reporting sustained long-term clinical symptomatic relief of RLS [10].

While these studies underscore the potential benefits of interventions such as superficial venous ablation and ultrasound-guided foam sclerotherapy in improving RLS symptoms, the mechanisms underlying this association merit further exploration. Previous research has suggested that venous insufficiency may contribute to neuronal dysfunction, potentially through alterations in blood flow dynamics, leading to an increased susceptibility to RLS symptoms [22,23]. Future investigations should consider neuroimaging and neurophysiological assessments to elucidate the specific pathways through which venous disorders influence the manifestation of RLS.

Sundaresan *et al*. expanded the scope by retrospectively reviewing leg vein treatments for RLS, highlighting the potential effectiveness of interventions such as radiofrequency ablation and USGFS [12]. This echoes existing literature suggesting that correction of superficial venous reflux may contribute to the improvement of RLS symptoms, emphasizing the need for a tailored approach in managing patients with concurrent venous and neurological symptoms [24,25].

Contrastingly, the study by Yetkin *et al*. did not find a significant association between peripheral varicose veins and RLS, raising questions about the heterogeneity of the relationship across different venous pathologies [13]. While this study provides valuable information about the prevalence of venous leg symptoms in the peripheral varicose vein group, the absence of a significant association prompts consideration of potential confounding factors. Factors such as the severity of venous insufficiency, specific characteristics of RLS symptoms, or the presence of comorbidities may influence the observed relationship. The comprehensive exclusion criteria in this study indicate the importance of accounting for confounding variables in future research exploring the link between RLS and venous disorders [13].

The study by Yolgösteren *et al*. highlights the bidirectional relationship between RLS and chronic venous insufficiency in the context of sleep disorders [14]. The coexistence of these conditions suggests a potential synergistic effect, with sleep disturbances exacerbating venous symptoms and vice versa. This finding aligns with existing literature emphasizing the impact of sleep disturbances on both the severity and quality of life in individuals with RLS [26-30]. The bidirectional relationship underscores the importance of comprehensive assessments that consider both neurological and venous factors in clinical practice [31].

McDonagh *et al*. further explored the prevalence of cramps in RLS-positive subjects with concurrent chronic venous insufficiency, shedding light on a potential subgroup of patients with a more advanced form of venous disease [15]. This aligns with the growing recognition of the systemic nature of venous disorders and their impact on various physiological systems [6]. The higher prevalence of cramps in RLS-positive subjects with concurrent chronic venous insufficiency suggests a synergistic relationship, potentially influencing the severity and progression of both conditions. Future research could delve into the underlying pathophysiological mechanisms linking cramps, RLS, and chronic venous insufficiency.

Acir *et al*. contributed valuable insights into the holistic benefits of varicose vein surgery, extending beyond the resolution of venous symptoms to improvements in sleep quality [16]. The reported improvement in sleep quality after surgery emphasizes the potential role of addressing venous pathology in managing not only RLS symptoms but also associated sleep disturbances. This finding aligns with the broader literature highlighting the bidirectional relationship between sleep disturbances and venous disorders, emphasizing the need for a multidisciplinary approach in patient care [32,33].

Fronek *et al*. did not find a significant difference in patterns of superficial venous reflux in patients with nocturnal symptoms compared to those without, underscoring the complexity of the relationship between superficial venous reflux and nocturnal symptoms [17]. While this study did not reveal a direct association between specific patterns of venous insufficiency and nocturnal leg symptoms, it raises questions about the heterogeneity of venous disorders and their impact on the manifestation of RLS symptoms. The study underscores the need for a more nuanced understanding of the complex relationship between superficial venous reflux and nocturnal symptoms, acknowledging the multifactorial nature of RLS [17].

Ulaş *et al*. delves into the investigation of serum irisin levels in idiopathic RLS, offering a potential biochemical marker for exploring the pathophysiological mechanisms of RLS [18]. The elevated serum irisin level in patients with idiopathic RLS suggests a potential role of this adipocytokine in the regulation of motor and sensory functions. The study adds a novel dimension to the exploration of RLS pathophysiology, hinting at potential links between adipose tissue, muscle function, and the neurological manifestations of RLS. The findings prompt further research into the role of irisin and related biomarkers in understanding the molecular underpinnings of RLS [34–36].

The study by Hayes *et al*. scrutinized the effect of endovenous laser ablation on restless legs syndrome, highlighting a statistically significant improvement in symptoms after operative correction of superficial venous insufficiency [1]. The marked improvement in the International Restless Legs Syndrome (IRLS) score and the percentage of patients experiencing relief underscore the potential therapeutic impact of addressing venous insufficiency in RLS management. These findings align with previous research indicating a close relationship between venous pathology and neurological symptoms [37,38].

## Conclusion

The collective evidence from the discussed studies offers a multifaceted perspective on the association between RLS and venous disorders. While some studies emphasize the positive correlation and potential benefits of interventions, others reveal complexities in the relationship or the absence of a significant association in certain contexts. The bidirectional relationship between RLS and venous disorders suggests a need for a holistic approach in clinical practice, considering both neurological and venous factors. Future research should focus on elucidating the underlying mechanisms, refining diagnostic criteria, and exploring tailored interventions for patients with concurrent RLS and venous insufficiency. A comprehensive understanding of this intricate relationship will undoubtedly enhance the quality of care and outcomes for individuals grappling with the challenges posed by both RLS and venous disorders.
